# Acute viral bronchiolitis phenotype in response to glucocorticoid and bronchodilator treatment

**DOI:** 10.1016/j.clinsp.2024.100396

**Published:** 2024-06-05

**Authors:** Andressa Roberta Paschoarelli Chacorowski, Vanessa de Oliveira Lima, Eniuce Menezes, Jorge Juarez Vieira Teixeira, Dennis Armando Bertolini

**Affiliations:** Universidade Estadual de Maringá, Maringá, PR, Brazil

**Keywords:** Bronchiolitis, Treatment, Phenotype, Glucocorticoid, Bronchodilator, Respiratory syncytial virus

## Abstract

**Objective:**

To analyze whether infants admitted to hospital with Acute Viral Bronchiolitis (AVB), who received glucocorticoids and bronchodilators, and who had an atopic phenotype, spent less time in hospital and/or less time on oxygen therapy when compared to those who did not have the phenotype.

**Method:**

A cross-sectional, retrospective epidemiological study was developed with data from medical records of infants admitted to hospital due to AVB from 2012 to 2019 in a sentinel public hospital. It was verified that the frequency of prescription of glucocorticoids, bronchodilators and antibiotics. Length of stay and oxygen therapy duration were then compared in the group that used glucocorticoids and bronchodilators between those who had a personal or family history of atopy and those who did not. Subsequently, the length of hospital stay was compared among infants who received antibiotic therapy and those who did not.

**Results:**

Fifty-eight infants were included. Of these, 62.1 % received an antibiotic, 100 % a bronchodilator and 98.3 % a glucocorticoid. When comparing infants without a family history of atopy, those who received antibiotics had a longer hospital stay (*p* = 0.01).

**Conclusion:**

The presence of an atopic phenotype did not interfere with the length of stay and/or oxygen therapy duration of those who received bronchodilators and glucocorticoids. Increased length of stay of infants without a family history of atopy, who used antibiotics without evidence of bacterial co-infection, and the high frequency of prescription of non-recommended drugs call attention to stricter protocol implementation and professional training in AVB diagnosis and care.

## Highlights

• The frequency of prescription of medications not recommended for bronchiolitis is high.
• Atopic phenotype did not interfere with the response to bronchodilators and glucocorticoids.
• The lower the respiratory rate, the shorter the oxygen therapy duration for bronchiolitis.

## Introduction

Bronchiolitis is one of the most common illnesses of early childhood.^1^^,^^2^ It is responsible for a large number of hospital admissions with a direct and indirect impact on health expenditures for the family and society as a whole.^1^, ^2^, ^3^ The United States alone accounts for an average of 100,000 hospital admissions per year.^4^ In Brazil, in 2019, more than 45.000 children were admitted to hospital for bronchiolitis.^5^

Despite medical and technological advances in the last 2 decades, there has not been a substantial change in Acute Viral Bronchiolitis (AVB) treatment and in the mean length of hospital stay for these patients.^6^ Since 2011, the Brazilian Society of Pediatrics (SBP) in 2011,^7^ through the publication of their guidelines, recommended that infants with AVB should only receive clinical management: oxygen therapy, ventilatory support, and hydration according to infants’ individual needs. The latest updates of Brazilian and American guidelines, from 2014 accordingly do not recommend using systemic glucocorticoids and bronchodilators as well as carrying out therapeutic tests with bronchodilators, considered in previous years. However, AVB clinical course and severity are quite variable, and recent studies indicate that bronchiolitis is a heterogeneous disease and that some patients could benefit from using these drugs.^3^^,^^9^, ^10^, ^11^, ^12^

In 2016, Dumas et al. identified different phenotypes of infants admitted to the hospital for AVB: Profile A ‒ Patient treated at the emergency service with wheezing, with a previous history of wheezing and eczema, who showed a greater association with Rhinovirus (RV) infection; Profile B ‒ Similar to Profile A, however without a previous history of bronchospasm or eczema, with a more prevalent Respiratory Syncytial Virus (RSV) infection; Profile C ‒ Moderate-severe respiratory effort, which required a longer hospital stay; Profile D ‒ The group in which patients did not wheeze, who had less severe disease. As a consequence, they suggest a potential difference in the response to the acute treatment and an important implication in the long-term outcome, with future risk for asthma.^1^

Rodríguez-Martínez et al., in a review study, describe phenotypes of infants with AVB that are more likely to have a positive response to bronchodilator use: older infants with AVB by RV, especially those with predominant *Haemophilus influenzae* in their microbiome nasopharyngeal; those affected during the months opposite the months of peak RSV; infants who have wheezing; those with a history of atopic dermatitis or a family history of asthma in a first-degree relative.^3^

Considering the emergence of this new knowledge regarding disease heterogeneity and the scarce national production on the frequency with which non-recommended drugs are prescribed in the present environment, this study aimed to analyze: how often infants admitted to hospitals with AVB received bronchodilators, glucocorticoids, and antibiotics; how to assess whether those who received glucocorticoids and bronchodilators and had an atopic phenotype spent less time in hospital and/or less time on oxygen therapy when compared to those who did not have the phenotype. It was chosen to test this response to bronchodilators and glucocorticoids in this specific group of patients due to the clinical similarity of AVB with asthma. Thus, a hypothesis was raised that patients with an atopic phenotype may benefit from using bronchodilators and glucocorticoids, presenting a shorter length of hospital admission and oxygen therapy duration, which corroborates the idea of individualizing the therapeutic approach in AVB guided by anamnesis. To ensure that antibiotic use did not constitute an isolated factor that increased the length of hospital stay, the present study's hypothesis was further tested for these subgroups (those who received and those who did not receive antibiotic therapy).

## Materials and methods

### Study design and place

An epidemiological, retrospective, cross-sectional study was carried out, in which patients under 2 years old admitted with a diagnosis of AVB at the *Hospital Universitário Regional de Maringá* (HUM), a tertiary public hospital, were identified through a search of medical records from 2012 to 2019. The study was conducted following the guidelines outlined in the Strengthening the Reporting of Observational Studies in Epidemiology (STROBE) statement.

### Study population

The numbers of physical records were obtained in two ways. The first corresponded to the period from 2012 to 2014, when the computerized system had not yet been implemented in the hospital, through a search in a book of hospital admission records in the ward or Pediatric Intensive Care Unit (ICU). The search was performed by diagnoses of bronchiolitis, acute respiratory failure, and asthma. In the second, from 2015 to 2019, through the SUS Health Care Management System (GSUS ‒ *Sistema de Gestão da Assistência de Saúde do SUS*), the International Classification of Diseases (ICD) was researched: J21, J96 e J45. It was decided to add the other diagnoses in the initial survey in addition to bronchiolitis, considering the possibility of diagnosing bronchiolitis among the alternative ICD.

### Variables of interest

The following were investigated in the medical records: age (days); sex; weight; gestational age at birth; comorbidities; final diagnosis (in discharge summary); the presence of wheezing; history of fever measured or reported by a caregiver; family history of atopy (father, mother or siblings with a history of allergic rhinitis, asthma or atopic eczema); history of atopy (previous wheezing, atopic eczema, allergic rhinitis); Exclusive Breastfeeding (EBF) duration; data from the first blood count; length of stay; and oxygen therapy. It also verified the prescription of antibiotics, systemic glucocorticoids and inhaled bronchodilators during the hospital admission period. Regarding vital signs (Heart Rate [HR], Respiratory Rate [RR] and Oxygen Saturation [SpO_2_]) in order to mitigate the effect of body temperature on other signs, the first set with a temperature below 37.5 °C was considered from admission. Afterward, the score of vital signs in pediatrics (Vipe)^13^ was used, which attributed a score to each sign according to infants’ age, and the sum of this score was categorized into one of the 5 groups of score severity (blue, green, yellow, orange and red - from least to most severe).

### Exclusion criteria

**Fig 1:**
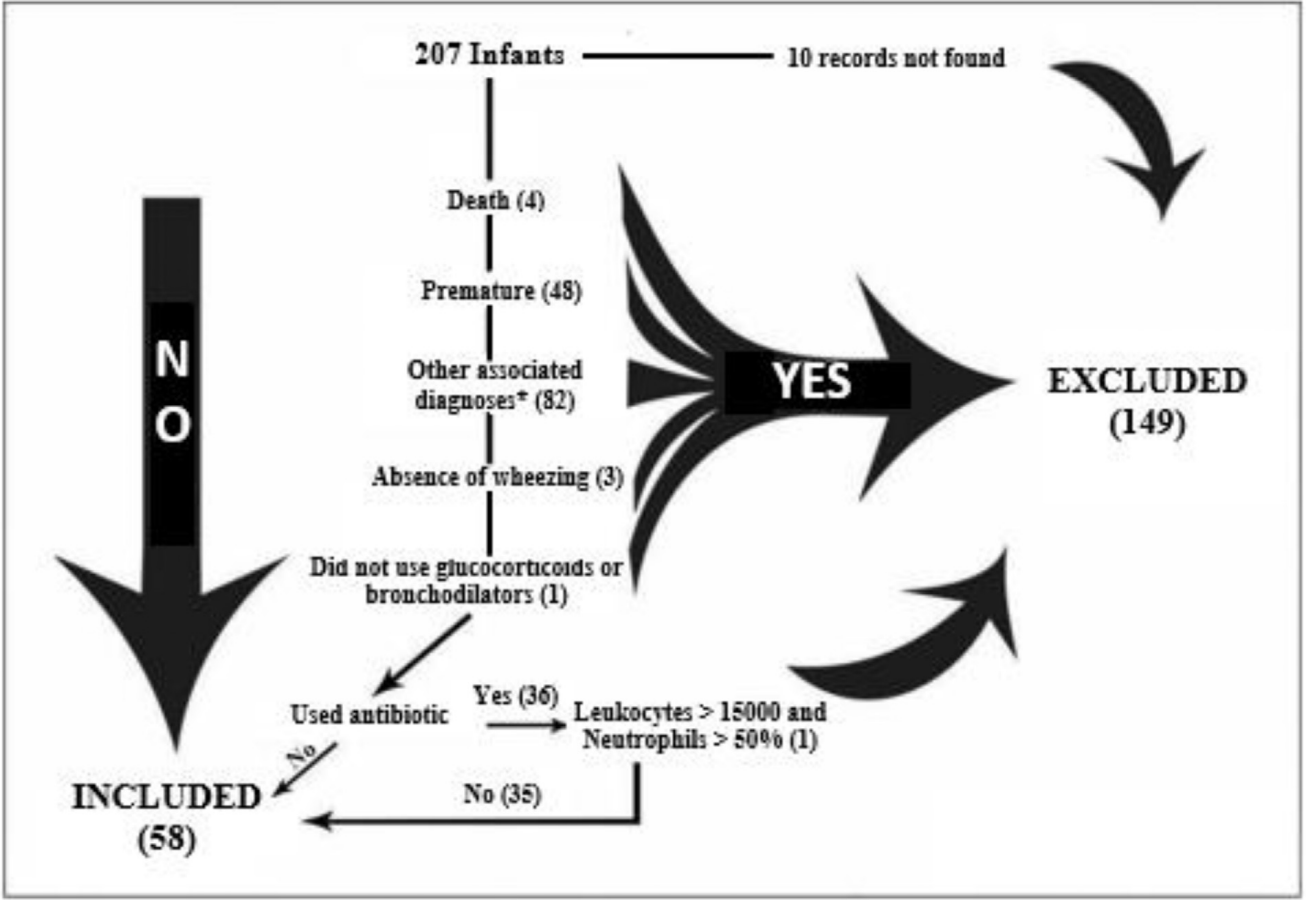
**Excluded infant flowchart.** *Other diagnoses: pneumonia (questioned or confirmed), chronic lung disease, genetic syndromes, neuropathy, heart disease, pertussis syndrome, and suspected tuberculosis.

Those patients who did not present a diagnosis of AVB in the discharge summary (which is the definitive admission diagnosis), premature individuals (less than 37 weeks), and those with other associated diagnoses, such as pneumonia, genetic syndromes, heart disease, pneumopathy, neuropathies, pertussis syndrome and patients who died (Fig. 1). Infants who did not present with wheezing at any point during hospital admission were also excluded. Among infants who received antibiotic therapy, those who presented leukocytes above 15,000 with a number of neutrophils above 50 % of the total leukocytes were excluded. The choice of this criterion was based on laboratory criteria present in protocols for fever without localizing signs in infants, which searches for bacterial disease in children under 36 months of age.^14^

After calculating the frequency of use of bronchodilators and systemic glucocorticoids, one infant who had not received glucocorticoids was also excluded, with no need to be excluded due to non-use of bronchodilators (100 % received them) (Fig. 1).

### Statistical analysis

Wilcoxon's non-parametric hypothesis test was used with a significance level of 5 % to test the association of length of stay and oxygen therapy duration separately with history of atopy, and family history of atopy. This analysis was also performed in the subgroup that received antibiotic therapy, comparing it with the group that did not receive it, both in the atopic group and in the group without atopy. Afterward, the association test was applied to compare the length of stay/oxygen therapy duration in the group that received antibiotics with the group that did not, regardless of personal or family history of atopy. The association between length of stay and/or oxygen therapy duration with sex, fever, Vipe score, HR, SatO_2_, RR, and Exclusive Breastfeeding (EBF) duration was also tested. Data were stored in Microsoft Excel® version 2211 and analyzed in R® version 4.1.2 (2021–11–01).

### Research ethics committee

This study was submitted to the Standing Research Ethics Committee Involving Human Beings (COPEP) of the *Universidade Estadual de Maringá*, and was approved on December 23, 2021 under *Certificado de Apresentação para Apreciação Ética* (CAAE ‒ Certificate of Presentation for Ethical Consideration) n° 54,236,221.2.0000.0104 (Opinion 5.186.320). Since it is secondary data, there was a waiver of the Informed Consent Form (ICF).

## Results

Thus, 207 medical records of individuals younger than two years were selected, but 149 were excluded (Fig. 1). Of the 58 infants included in the study, 32 (55.2 %) were male. The median age was 109 days, with 18 infants over 180 days old and only 5 over one year old. According to the parameters adopted by the Ministry of Health,^15^ 52 (89.4 %) infants were classified as having adequate weight for their age (Table 1).

**Table 1:** Clinical and epidemiological characteristics of infants treated at the *Hospital Regional Universitário de Maringá* from 2012 to 2019.

**Age (days)**	**n (%)**
0‒60	17 (29.3)
61‒180	23 (39.7)
181‒365	13 (22.4)
> 365	5 (8.6)
**Mean:**	**144.9 days**
**Exclusive Breastfeeding (EBF) duration in those over 180 days**	**n (%)**
Still in EBF^a^	1 (5.5)
Less than 30 days	1 (5.5)
30‒119 days	8 (44.4)
120‒180 days	2 (11.1)
Undetermined	6 (33.3)
**Nutritional (z-score)**	**n (%)**
High weight for age (> +2)	1 (1.8)
Adequate weight for age (≥ −2 and ≤ +2)	51 (89.4)
Low weight for age (< −2 and ≥ −3)	4 (7.0))
Very low weight for age (< −3)	1 (1.8)
**Length of hospital stay (days)**	**n (%)**
0‒3	17 (33.3)
4‒6	16 (31.3)
7‒10	15 (29.4)
+ than 10	3 (6)
**Mean:**	**5.4± 3.2**
**ICU length of stay (days)**	**n (%)**
1‒2	12 (57.1)
3‒4	5 (23.8)
5‒6	3 (14.3)
7 or more	1 (4.8)
**Mean**:	**2.6 ± 1.9**
**Oxygen therapy duration**	**n (%)**
Did not use	13 (22.4)
Less than 24 h	4 (7)
Undetermined	5 (8.6)
1‒2 days	22 (37.9)
3‒4 days	9 (15.5)
More than 4 days	5 (8.6)
**Mean among those who used more than 24 h:**	**2 days**
**VIP score**	**n (%)**
Blue (0)	5 (9.6)
Green (1‒2)	6 (11.5)
Yellow (3‒5)	14 (26.9)
Orange (6‒9)	26 (50.0)
Red (≥ 10)	1 (1.9)
**History of atopy (eczema, previous wheezing, allergic rhinitis)**	**n (%)**
Yes	5 (8.6)
No	44 (75.9)
Undetermined	9 (15.5)
**Family history of atopy (eczema, asthma, allergic rhinitis)**	**N (%)**
Yes	22 (37.9)
No	24 (41.4)
Undetermined	12 (20.7)

a 203-days.

The mean length of stay was 5.4 days (±3.2). This calculation was only possible with 50 infants, since, after discharge from the ICU, 8 patients returned to the hospital of origin, where they completed the length of stay. Twenty-one infants (36.2 %) were admitted to the ICU with a mean of 2.6 days (standard deviation of ±1.9) of stay in this sector. No patient required mechanical ventilation, and 43 (74.1 %) infants received oxygen therapy at some point. Of these, 4 received oxygen for less than 24 h. The mean time of those who received oxygen for more than 24 h was 2 days (variance 5.4 ± 2.3 days) (Table 1).

All patients received systemic glucocorticoids and inhaled bronchodilators within the first 24 h of admission, and 62.1 % (36/58) received some antibiotic for at least 2 days, starting within 24 h of admission: 21 received azithromycin (58 % of those who used antibiotics); 3 received ceftriaxone; 3 received crystalline penicillin; 3 received ampicillin; and 1 used amoxicillin as sole antibiotic therapy. Among the infants, 5 received 2 antibiotics (4 azithromycin and ampicillin or ceftriaxone or crystalline penicillin), and 1 received ampicillin and gentamicin.

Of the infants older than 180 days, 12 had information on EBF duration, and 66.7 % (8) of these were breastfed from 30 to 119 days. There was no influence of EBF duration on length of hospital stay or oxygen therapy duration (*p* = 0.60; 0.23) (Table 2).

**Table 2:** Comparison of variables regarding length of stay and oxygen therapy duration in infants admitted to the *Hospital Regional Universitário de Maringá* from 2012 to 2019.

**Variables**	**Length of hospital stay (n)**	**p**	**Oxygen therapy duration (n)**	**p**
**EBF**				
≥ 120 days	8	0.6	5	0.23
< 120 days	2		2	
**SpO_2_**				
≤ 1	16	0.14	14	
> 1	20		21	0.26
**HR**				
≤ 1	10	0.66	11	
> 1	29		26	0.20
**RR**				
≤ 1	22		21	**0.03**
> 1	17	0.82	16	
**Vipe score**				
≤ 1	4	0.96	4	
> 1	32		31	0.10
**History of atopy**				
With a history of atopy	4		3	
No history of atopy	36	0.57	34	0.73
**Family history of atopy**				
With a family history of atopy	20		17	
No family history of atopy	20	0.46	20	0.34
**Fever**				
With fever	26		19	
No fever	14	0.69	18	0.36
**Sex**				
Male	22		25	
Female	18	0.76	12	0.97
**Antibiotic**				
With antibiotic	26	0.07	25	
No antibiotic	14		12	0.06

EBF, Exclusive Breastfeeding; SpO_2_, Oxygen Saturation; HR, Heart Rate; RR, Respiratory Rate; Vipe, Vipe Score.

Vital signs were described for 52 (89.7 %) infants, and 58.6 % (34) reported fever at some point during the illness. Of these, 26 (50.0 %) received orange stratification by the Vipe score (Table 1). No change in vital signs, tested for different cut-off points, was associated with length of stay, and only RR was associated with oxygen therapy duration (*p* = 0.03), in which infants with RR with a score ≤1 received oxygen therapy for less time (Table 2).

As for the history of atopy, 5 (8.6 %) patients had a history of eczema or allergic rhinitis or a previous episode of wheezing, 9 (15.5 %) with undetermined data (no information in the medical record). Twenty-two (47.8 %) had first-degree relatives with a history of atopy (Table 1). There was no statistically significant difference in the length of hospital stay or oxygen therapy duration when considering the atopic phenotype ‒ personal or family (Table 2). When infants without a family history of atopy were compared to each other, those who had received antibiotics had a longer hospital stay than those who did not (*p* = 0.01) (Table 3). In general, those who received antibiotics tended to remain admitted to the hospital longer than those who did not (*p* = 0.07) (Table 2).

**Table 3:** Comparison of length of stay of infants admitted to the *Hospital Universitário Regional de Maringá* with a history of atopy and a family history of atopy with infants without a history in the subgroups that received antibiotics or not from 2012 to 2019.

**Group**	**Received antibiotic**	**Family history of atopy and history of atopy**		**p**
		**Yes**	**No**	
Group 1B	**Yes**	2		1
Group 2B	**No**	2		
				
Group 1B	**Yes**	2		0.74
Group 3B	**Yes**		10	
				
Group 1B	**Yes**	2		0.16
Group 4B	**No**		10	
				
Group 3B	**Yes**		10	0.83
Group 2B	**No**	2		
			
Group 4B	**No**		10	0.75
Group 2B	**No**	2		
				**0.01**
Group 3B	**Yes**		10	
Group 4B	**No**		10	

Wilcoxon test: Group 1B (received antibiotics and have a personal and family history of atopy); Group 2B (did not receive antibiotics and have a personal and family history of atopy); Group 3B (received antibiotics and no personal or family history of atopy); Group 4B (did not receive antibiotics and has no personal or family history of atopy).

## Discussion

The present study did not show any difference in length of hospital stay or oxygen therapy duration of infants with AVB who received systemic glucocorticoids and bronchodilators, when considering the phenotype as follows: presence or absence of a personal history of atopy or a family history of atopy. Another highlight was the high frequency of prescription drugs not recommended by national guidelines.^9^

Despite the recognized advances in understanding AVB that have occurred in recent decades, it has not yet witnessed a substantial change in AVB treatment and in the mean length of stay of these patients.^6^^,^^16^^,^^17^ Kirolos et al. (2019), in a systematic review, compared the recommendations of 32 guidelines on AVB published until 2017. Of these, 22 recommend against using bronchodilators, 14 consider the findings controversial or consider the therapeutic test in specific situations, and only 3 recommend its use. Glucocorticoids as well as antibiotics are not recommended by any, and the non-recommendation of using glucocorticoids is present in 29 (90.6 %) of the 32 guidelines.^18^ However, in recent years, some authors have defended the hypothesis that AVB is a heterogeneous condition and that treatment should be individualized, considering a patient's specific phenotype.^2^^,^^19^, ^20^, ^21^, ^22^ Thus, a group of patients could benefit from using medications that, until now, have not been recommended.^9^^,^^11^^,^^18^^,^^23^ Megalaa et al. (2018), for instance, suggest that infants with asthma, mistakenly diagnosed with AVB, could benefit from using glucocorticoids and bronchodilators.^20^

In this line, a Pakistani multicenter study with 212 infants compared the clinical response between patients with a family history of atopy and patients without a family history to prednisolone use in the face of AVB and observed a shorter hospital stay and a significant improvement in symptoms among patients with a family history of atopy,^19^ which is in contrast to the present findings. This difference can be explained by the difference in the predominant age group in the studies ‒ the infants had a lower mean age, with the majority (68.9 %) being younger than 6 months (mean 4.8 ± 4), while in the Pakistani study, the majority was older than 6 months (mean 5.39±3.11), with a lower age of inclusion in the study of 2 months. Rodríguez-Martínez (2021) suggests, in his review, that infants with AVB older than 6 months and with a parental history of asthma may be potential responders to bronchodilators.^3^

A randomized clinical trial conducted in Qatar found shorter hospital stays for infants with AVB who received oral dexamethasone and salbutamol and had risk factors for asthma such as a history of eczema or a family history of eczema or asthma (OR = 0.69, 95 % CI 0.51 to 0.93) when compared to infants without risk factors.^24^ This study, unlike ours, considered preterm infants older than 34 weeks, who may have an immune response and clinical behavior different from infants exclusively older than 37 weeks.^25^

Different tools for severity categorization were used in the studies from Pakistan, Qatar, and the present study: Modified Respiratory Distress Assessment Instrument (mRDAI) Score, Wang Bronchiolitis Severity Score and Vipe score, respectively.^13^^,^^26^^,^^27^ Although the Vipe score only considers vital signs and does not compare respiratory effort data, the choice of this severity categorization tool is justified as it considers objective data routinely documented in medical records and allows comparing RR between different age groups, which is not included in other scores.^26^^,^^27^ Furthermore, the Vipe score uses age segmentation for reference values of vital signs, as recommended by the World Health Organization: 0‒2, 3‒11, 12‒24 months.^28^ Additionally, the present analysis resulted in the association of the Vipe score for RR ≤ 1 for age (Table 2) with shorter oxygen therapy time (*p* = 0.03), this variable being data already related to the clinical evolution of pediatric patients.^29^

Mansbach et al. (2016), in a multicenter study, showed that infants admitted to the hospital due to AVB caused by RV have phenotypic characteristics of asthmatic infants (older infants, with a history of wheezing or a history of eczema) when compared to AVB caused by RSV.^12^ Sena et al. (2021) identified a higher risk of asthma in infants, and children of an asthmatic mother, with AVB via RV.^30^ Lukkarinen et al. (2013), in a randomized, double-blind study, found benefit in using prednisolone by patients admitted to the hospital for AVB caused by RV and/or with a history of eczema, in reducing recurrent wheezing.^31^ Martínez et al. (2022) associated the decrease in using bronchodilators with earlier hospital discharge, which could save material and human resources.^32^

Using intravenous antibiotics has already been described in other works as an isolated risk factor for increasing children's length of stay.^33^^,^^34^ In the present study, there was a tendency towards an increase in the length of hospital stay related to using antibiotics, regardless of history of atopy (*p* = 0.07). When considering a family history of atopy, infants without a history who received antibiotics remained admitted to the hospital longer than those who did not (*p* = 0.01). This result probably did not extend to the other analyses due to the small sample values of the assessed subgroups, which is one of the limitations of the present study. Another finding that corroborates a possible isolated influence of using antibiotics on length of stay was that it was not found a longer period of oxygen therapy in the group that had a longer length of stay associated with antibiotic use, i.e., this infant's clinical evolution did not differ between the groups (with and without antibiotics). This influence on length of stay can be explained by the fact that the attending physician wants to guarantee complete treatment of these infants in a hospital environment, either due to insufficient trust in caregivers or due to the difficulty of accepting oral medications presented by some babies, requiring a complete intravenous treatment.

The frequency with which drugs not recommended by Brazilian guidelines were prescribed^8^ is much higher than that found in the literature as a practice in other countries (Table 4). The most prescribed antibiotic was azithromycin, which, despite the already described immunomodulatory effect of macrolides,^35^ does not present data that justify its use in AVB.^32^ This high frequency of antibiotic prescription is probably due to clinical concerns about co-infections and complications in cases of severe AVB.

**Table 4:** Comparative values of the frequency of prescription of bronchodilator, glucocorticoid and antibiotic for patients with AVB between different studies of different nationalities.

**Country**	**Bronchodilator (%)**	**Glucocorticoid (%)**	**Antibiotic (%)**
**Brazil – 2022**	**100**	**98.3**	**62**
Italy-Manti S*.* et al., 2021^37^	39.6	64.5	4.7
Australia and New Zealand – Oakley S. et al., 2018^38^	24.2	11.6	14.1
Jordan-Awad S. et al., 2020^39^	31.9	5.5	16.5
Spain-Martinez D.A. et al., 2022^32^	51.2	‒	‒

A study published in 2017 with 38 hospitals from 8 countries showed that, on average, 14 % of infants with AVB received a bronchodilator and 2.3 % a glucocorticoid.^36^ In Brazil, there are few studies on the frequency with which these drugs are prescribed for AVB. Despite the result portraying the medical conduct in a single service, it is a tertiary public hospital, with a sentinel profile for surveillance of respiratory viruses. It is suggested, however, that multicenter studies be carried out involving hospitals in the state of Paraná, for greater robustness and external evidence validity.

Other limitations that can be mentioned in this study are the nature of the data and the incomplete filling of medical records and the non-identification of viruses for analysis. Moreover, the sample was reduced as a result of strict exclusion criteria in an attempt to avoid any bias, such as concomitant bacterial infection, which could influence the present results. Despite the limitations, the study provides useful data to corroborate results that guide the main guidelines for AVB treatment in the world, when considering a family history of atopy or a history of atopy in infants.

It was concluded that the presence of an atopic phenotype did not interfere with the length of stay and/or oxygen therapy duration of infants with AVB who received inhaled bronchodilators and systemic glucocorticoids in the same way as breastfeeding duration. Among the vital signs, only RR was related to oxygen therapy duration. It was highlighted here an increased length of stay for infants without a family history of atopy who used antibiotics when compared to those who did not. Until new studies are carried out considering the phenotype for AVB treatment, guidelines based on clinical evidence proposed by current guidelines should guide the clinical practice of professionals who assist this population. Better strategies are needed to promote stricter protocol implementation and professional training in AVB diagnosis and care. This will be reflected in the reduction of expenses with drugs that do not offer a proven benefit, also reducing the frequency of their side effects and patient's length of stay.

## CRediT authorship contribution statement

**Andressa Roberta Paschoarelli Chacorowski:** Conceptualization, Investigation, Data curation, Methodology, Writing – original draft. **Vanessa de Oliveira Lima:** Formal analysis. **Eniuce Menezes:** Methodology. **Jorge Juarez Vieira Teixeira:** Conceptualization, Supervision, Writing – review & editing. **Dennis Armando Bertolini:** Project administration, Supervision, Writing – review & editing.

## Declaration of competing interest

The authors declare no conflicts of interest.

## Appendix Supplementary materials

Image, application 1
