# Supplementary material for: Acute viral bronchiolitis phenotype in response to glucocorticoid and bronchodilator treatment
Source: Clinics (Sao Paulo). 2024 Jun 5;79:100396. doi: 10.1016/j.clinsp.2024.100396 (PMC11215958; doi:10.1016/j.clinsp.2024.100396)

**CLINICS-D-23-00711_Supplementary Material**

**Table** **Supplementary** **1** Calculation of the Vipe Score (adapted from Magalhães-Barbosa MC et al., 2016) in infants admitted to the *Hospital Regional Universitário de Maringá*, from 2012 to 2019.

| **Newborn to 2 full months** | | | | | | | |
| --- | --- | --- | --- | --- | --- | --- | --- |
|  | **4** | **2** | **1** | **0** | **2** | **3** | **4** |
| RR | < 16 | 16‒19 | 20‒29 | 30‒60 | 61‒80 | 81‒90 | > 90 |
| HR | < 81 | 81‒90 | 91‒110 | 111‒149 | 150‒179 | 180‒189 | > 189 |
| SpO_2_ | < 90 | 90‒92 | 93‒94 | 95‒100 |  |  |  |
|  |  |  |  |  |  |  |  |
| **3 months to 11 full months** | | | | | | | |
|  | **4** | **2** | **1** | **0** | **2** | **3** | **4** |
| RR | < 16 | 16‒19 | 20‒24 | 25‒50 | 51‒70 | 71‒80 | > 80 |
| HR | < 71 | 71‒90 | 81‒100 | 101‒139 | 140‒169 | 170‒179 | > 179 |
| SpO_2_ | < 90 | 90‒92 | 93‒94 | 95‒100 |  |  |  |
|  |  |  |  |  |  |  |  |
| **1 year to 2 years** | | | | | | | |
|  | **4** | **2** | **1** | **0** | **2** | **3** | **4** |
| RR | < 13 | 13‒15 | 16‒19 | 20‒40 | 41‒60 | 61‒70 | > 70 |
| HR | < 61 | 61‒70 | 71‒90 | 91‒119 | 120‒149 | 150‒169 | > 149 |
| SpO_2_ | < 90 | 90‒92 | 93‒94 | 95‒100 |  |  |  |
|  |  |  |  |  |  |  |  |
| **Risk stratification = sum of scores: RR+HR+ SpO_2_** | | | | | | | |
|  |  |  |  |  |  |  |  |
| Blue | 0 |  |  |  |  |  |  |
| Green | 1‒2 |  |  |  |  |  |  |
| Yellow | 3.5 |  |  |  |  |  |  |
| Orange | 6‒9 |  |  |  |  |  |  |
| Red | ≥ 10 |  |  |  |  |  |  |

**Table Supplementary 2** Comparison of variables regarding length of stay and oxygen therapy duration in infants admitted to the *Hospital Regional Universitário de Maringá*, from 2012 to 2019.

| **Variables** | **Length of hospital stay (n)** | **p** | **Oxygen therapy duration (n)** | **p** |
| --- | --- | --- | --- | --- |
| **EBF** |  |  |  |  |
| ≥ 120 days | 8 | 0.6 | 5 | 0.23 |
| < 120 days | 2 |  | 2 |  |
| **SpO_2_** |  |  |  |  |
| ≤ 1 | 16 | 0.14 | 14 |  |
| > 1 | 20 |  | 21 | 0.26 |
| **HR** |  |  |  |  |
| ≤ 1 | 10 | 0.66 | 11 |  |
| > 1 | 29 |  | 26 | 0.20 |
| **RR** |  |  |  |  |
| ≤ 1 | 22 |  | 21 | **0.03** |
| > 1 | 17 | 0.82 | 16 |  |
| **Vipe score** |  |  |  |  |
| ≤ 1 | 4 | 0.96 | 4 |  |
| > 1 | 32 |  | 31 | 0.10 |
| **History of atopy** |  |  |  |  |
| With a history of atopy | 4 |  | 3 |  |
| No history of atopy | 36 | 0.57 | 34 | 0.73 |
| **Family history of atopy** |  |  |  |  |
| With a family history of atopy | 20 |  | 17 |  |
| No family history of atopy | 20 | 0.46 | 20 | 0.34 |
| **Fever** |  |  |  |  |
| With fever | 26 |  | 19 |  |
| No fever | 14 | 0.69 | 18 | 0.36 |
| **Sex** |  |  |  |  |
| Male | 22 |  | 25 |  |
| Female | 18 | 0.76 | 12 | 0.97 |
| **Antibiotic** |  |  |  |  |
| With antibiotic | 26 | 0.07 | 25 |  |
| No antibiotic | 14 |  | 12 | 0.06 |

EBF, Exclusive Breastfeeding; SpO_2_, Oxygen Saturation; HR, Heart Rate; RR, Respiratory Rate; Vipe, Vipe score.

**Table Supplementary 3** Comparison of length of stay of infants admitted to the *Hospital Universitário Regional de Maringá* with a history of atopy or a family history of atopy (analyzed separately) with infants without a history in the subgroups that received antibiotics or not, from 2012 to 2019.

| **Group** | **Received antibiotic** | **Family history of atopy** | | **p** | **History of atopy** | | **p** |
| --- | --- | --- | --- | --- | --- | --- | --- |
|  |  | **Yes** | **No** |  | **Yes** | **No** |  |
| Group 1A | Yes | 16 |  | 0.39 | 2 |  | 0.46 |
| Group 2A | Yes |  | 10 |  |  | 24 |  |
|  |  |  |  |  |  |  |  |
| Group 1A | Yes | 16 |  | 0.08 | 2 |  | 0.23 |
| Group 3A | No |  | 10 |  |  | 12 |  |
|  |  |  |  |  |  |  |  |
| Group 1A | Yes | 16 |  | 0.81 | 2 |  | 1 |
| Group 4A | No | 4 |  |  | 2 |  |  |
|  |  |  |  |  |  |  |  |
| Group 2A | Yes |  | 10 | **0.01** |  | 24 | 0.08 |
| Group 3A | No |  | 10 |  |  | 12 |  |
|  |  |  | |  |  |  |  |
| Group 2A | Yes |  | 10 | 1 |  | 24 | 0.77 |
| Group 4A | No | 4 |  |  | 2 |  |  |
|  |  |  |  |  |  |  |  |
| Group 3A | No |  | 10 | 0.2 |  | 12 | 0.85 |
| Group 4A | No | 4 |  |  |  | 2 |  |

Wilcoxon test: Group 1A (received antibiotics and have a family history of atopy); Group 2A (received antibiotics and no family history of atopy); Group 3A (did not receive antibiotics and have no family history of atopy); Group 4A (did not receive antibiotics and has a family history of atopy).

**Figure Supplementary 1** Flowchart of statistical analyzes performed (Wilcoxon test) considering family history of atopy in infants admitted to the *Hospital Regional Universitário de Maringá*, from 2012 to 2019.


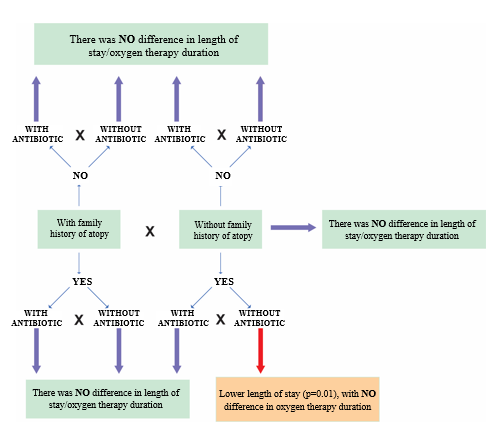


**Figure Supplementary 2** Antibiotics used by infants hospitalized with AVB at the *Hospital Regional Universitário de Maringá*, from 2012 to 2019.


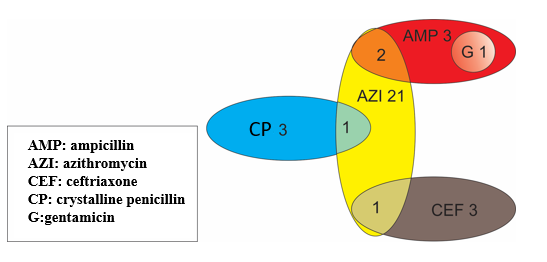

Supplement: Supplementary file 1 [file mmc1.docx]
